# A single-cell lung atlas of complement genes identifies the mesothelium and epithelium as prominent sources of extrahepatic complement proteins

**DOI:** 10.1038/s41385-022-00534-7

**Published:** 2022-06-07

**Authors:** Neha Chaudhary, Archana Jayaraman, Christoph Reinhardt, Joshua D. Campbell, Markus Bosmann

**Affiliations:** 1 Pulmonary Center, Department of Medicine, Boston University School of Medicine, Boston, MA USA; 2 Center for Thrombosis and Hemostasis, University Medical Center Mainz, Mainz, Germany; 3 Division of Computational Biomedicine, Boston University School of Medicine, Boston, MA USA

## Abstract

To understand functional duality of the complement system in host defense and lung injury, a more comprehensive view of its localized production in the lung, and the impact of age on complement production are essential. Here, we explored the expression of complement genes through computational analysis of preexisting single cell RNA sequencing data from lung transcriptomes of healthy young (3 months) and old C57BL/6 mice (24 months), and humans. We characterized the distribution of 48 complement genes. Across 28 distinct immune and non-immune cell types in mice, mesothelial cells expressed the greatest number of complement genes (e.g., *C1ra*, *C2, C3*), and regulators (e.g., *Serping1*, *Cfh*). *C5* was abundant in type II alveolar epithelial cells and *C1q* in interstitial lung macrophages. There were only moderate differences in gene expression between young and old mice. Among 57 human lung cell types, mesothelial cells showed abundant complement expression. A few differences in gene  expression (e.g., *FCN1*, *CFI*, *C6*, *C7*) were also evident between mice and human lung cells. Our findings present a novel perspective on the expression patterns of complement genes in normal lungs. These findings highlight the potential functions of complement in tissue-specific homeostasis and immunity and may foster a mechanistic understanding of its role in lung health and disease.

## Introduction

Lungs are large barrier organs with an extensive mucosal surface and are characterized by remarkable and complex cellular heterogeneity. Innovations in omics-based technologies have facilitated the identification of new cell types^1,2^. A recent study reported as many as 57 distinct cell phenotypes in the human lung^3^. These cell types include various non-immune cell populations such as epithelial, endothelial, and mesenchymal/stromal cells. The prominent leukocyte populations are mononuclear phagocytes, lymphocytes, and rare megakaryocytes^3^. Each of these cell populations exhibit functional plasticity and are involved in multiple regulatory pathways.

Airway epithelial cells such as goblet and ciliated cells are required for mucociliary clearance of inhaled particles. Alveolar epithelial type 1 (AT1) cells provide a short-distance barrier for diffusion gas exchange^4^. AT2 cells maintain homeostasis, secrete surfactant, mediate defense against pathogens (including MHCII-dependent antigen presentation^5^), and serve as AT1 progenitors^4^. Pulmonary capillary endothelial cells facilitate gas exchange and leukocyte migration^6^. Other subsets of lung endothelial cells from the pulmonary lymphatics, pulmonary blood vessels, and systemic blood vessels in the lung await functional annotations^7^. Mesenchymal cells, including smooth muscle cells, fibroblasts and mesothelial cells, are involved in homeostasis, repair, and wound healing^8^. Mesothelial cells cover the pleural spaces, secrete glycosaminoglycans and surfactant, and initiate inflammatory responses to pathogens via pro-inflammatory mediators such as interleukins (e.g., IL-6, IL-8) and interferons^9,10^. Amongst myeloid cells, alveolar macrophages are potent airway scavengers and modulate the magnitude of immune responses and tissue homeostasis^11^. Interstitial macrophages, localized in the parenchyma, function in inflammation, wound healing, and tissue repair^11^. Monocytes patrol the blood and play a role in surveillance of endothelial integrity and are recruited to the inflamed lungs^12^. Dendritic cells activate naïve T-cells, initiating adaptive immunity^11^. Granulocytes such as neutrophils and eosinophils modulate immune response and are involved in pathogen killing and clearance^11^. Resident memory CD4^+^ and CD8^+^ T-cells function in adaptive immune responses through the secretion of cytokines, activation of macrophages and lung stromal cells, and/or elimination of infected cells^13^. Tissue resident B-cells and plasma cells mediate humoral immunity and pathogen clearance through antibody production^14,15^.

**Fig. 1:**
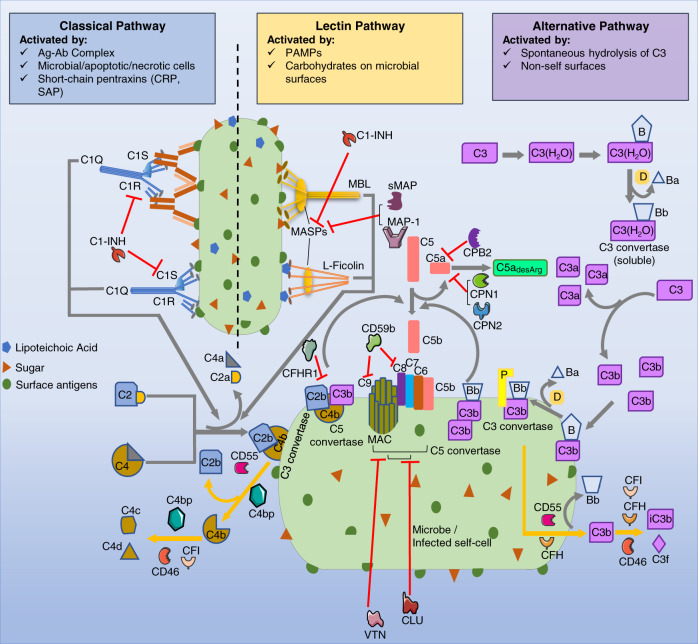
Overview of the complement pathways. The complement system is activated when its proteins encounter pathogens or damaged/infected self-cells and recognize a pattern on the surface of these cells. These events trigger the activation of complement through one of three pathways: the classical pathway (CP), the lectin pathway (LP) and the alternative pathway (AP). CP can be triggered by antigen-antibody (Ag-Ab) complexes, microbes, apoptotic cells, or short-chain pentraxins (e.g., C-reactive protein [CRP], serum amyloid P [SAP]) binding to the C1Q-C1R-C1S complex. This binding cleaves complement C4 and C2 to their respective peptide fragments and leads to the formation of the CP C3 convertase, C4b2b, and the C5 convertase (C4b2bC3b). LP is activated by attachment of mannose-binding lectin to cell surface carbohydrates on the pathogen and pathogen associated molecular patterns (PAMPs). This results in the MASP1 and MASP2 serine proteases, associated with the lectin pattern recognition molecules (MBL, ficolin), facilitating C3 convertase (C4b2b) and C5 convertase (C4b2bC3b) formation by cleaving C4 and C2 to peptide fragments. AP is activated in response to cellular damage and mediated by spontaneous hydrolysis of C3 or stimulation of C3 by properdin (P/CFP). The hydrolyzed C3 then forms a complex with factor B (B/CFB) and factor D (D/CFD) to form the AP C3 convertase (C3bBb). The CP, LP and AP C3 convertases can cleave C3 into C3a (anaphylatoxin) and C3b (opsonin) peptide fragments. Two C3b molecules can further associate with the factor B peptide Bb to form the AP C5 convertase C3bBb3b. The C5 convertases cleave C5 into C5a (anaphylatoxin) and C5b peptide fragments. The C5b fragment forms a membrane attack complex (MAC) together with C6–C9 complement component proteins that insert into the pathogen cell membrane, forming a pore and leading to cell lysis. C3a and C5a mediate phagocytosis, chemotaxis of immune cells and immunomodulation by binding to their respective G-protein coupled transmembrane receptors (C3AR1, C5AR1, C5AR2). Complement regulators such as CD46, CD55, CD59b (all membrane-anchored), C1-INH, sMAP, MAP-1, factor H (CFH), factor I (CFI) and carboxypeptidases (CPN1, CPN2, CPB2) closely monitor and modulate the complement activation and propagation^16,19,24^.

The proteolytic complement system is an essential part of the innate immune response to pathogens that infect the lung^16^. Activation of the complement system is mediated by one of three pathways: the classical pathway (CP) is potently initiated via the C1 protein complex through IgM/IgG antibodies bound to antigens, especially when clustered as pentameric or hexameric structural motifs^17^. The lectin pathway (LP) is triggered by attachment of mannose-binding lectin and other pattern recognition molecules (e.g., ficolins) to cell surface carbohydrates on the pathogen. The alternative pathway (AP) is activated in response to cellular damage on non-self surfaces (CD46^−^CD55^−^CD59^−^) either by spontaneous hydrolysis of effector C3 or activated as an amplification loop for the other pathways^18,19^ (Fig. 1; Supplementary Table 1). All of these events result in the formation of C3 convertases that cleave C3 into C3a (anaphylatoxin) and C3b (opsonin) fragments, and C5 convertases that cleave C5 into C5a and C5b with subsequent formation of the membrane attack complex (C5b-C9). These pathways involve regulated interactions amongst a network of nearly 50 complement proteins which can be classified as pattern recognition molecules, proteases, complement components, receptors, and regulators^18–20^.

Although the traditional view favors hepatic synthesis of inactive complement precursors, newer concepts appreciate localized production of complement genes in peripheral tissues and at mucosal surfaces such as the intestines (e.g., C3)^21^ and lung (e.g., C2-C5, factor B)^16,22^. However, a comprehensive analysis of the expression of complement genes in the context of the evolving cellular landscape of the lung is not available.

In the current study, we have profiled the expression of 48 complement genes in different lung cells through bioinformatics analysis of single cell RNA sequence data (scRNA-Seq) from healthy young and old mice (3 versus 24 months of age) generated by Angelidis et al.^23^, and from healthy tissue from human lungs by Travaglini et al.^3^.

Aberrant activation of the complement has been linked to the pathogenesis of age-associated diseases such as age-related macular degeneration^23^. However, the impact of age on complement production in different lung cell types has not been elucidated. Hence, we have also examined age-associated differences in the complement transcriptome profiles in immune and non-immune cell types in mice.

Overall, we observed that the classical and alternative pathways genes were expressed more compared with lectin pathway in both mice and humans, suggesting potential localized enforcement of complement activation through these pathways in the lung. Aging did not have a large impact on the expression of complement genes in the mouse dataset. These findings may provide a useful perspective in understanding cell type specific dysregulation of the complement system in the pathogenesis of lung diseases.

## Results

**Fig. 2:**
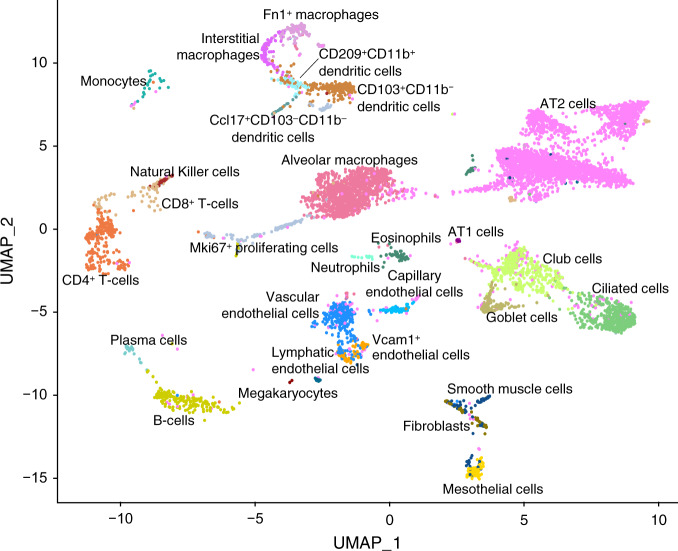
Visualization of different cell types identified in the mouse lung transcriptome. Uniform Manifold Approximation and Projection (UMAP) plot of lung single cell transcriptomes from young C57BL/6 mice (*n* = 7473 cells from 8 mice, 3 months old).

To elucidate the cell type-specific expression of complement genes in lung, while complying with IACUC guidelines of reducing the numbers of mice needed for research, we performed a search in NCBI’s Gene Expression Omnibus database for a dataset of untreated, healthy C57BL/6 mice. The scRNA-Seq study dataset by Angelidis et al.^23^ that included lung tissues from eight young mice (3 months) and seven old mice (24 months), was used in our analysis (Supplementary Fig. 1a). Quality control parameters such as unique molecular identifiers (UMI) per cell and percentage of mitochondrial genes were comparable across age groups (Supplementary Fig. 1b–i). After pre-processing and unsupervised clustering (see Methods for details), we categorized the dataset into 28 distinct cell types based on expression of markers from the original publication and literature^23^. The dataset comprised a wide variety of immune cells (e.g., alveolar macrophages, dendritic, T- and B-cells) and non-immune cells (e.g., AT1, AT2, ciliated, mesothelial, and endothelial cells) (Fig. 2; Supplementary Figs. 2 and 3). In digested lungs of both young and old mice, AT2 cells were most abundant, followed by alveolar macrophages, however their absolute numbers were higher in young mice (Supplementary Fig. 1j). A few cell types such as CD4^+^ and CD8^+^ T-cells, B-cells and plasma cells, ciliated cells, and monocytes were more abundant in old mice (Supplementary Fig. 1j).

We analyzed the cell type specific expression of 48 complement genes, which were classified in 5 groups as pattern recognition molecules, proteases, complement component genes, receptors and regulators^19^.

### Pattern recognition molecules

**Fig. 3:**
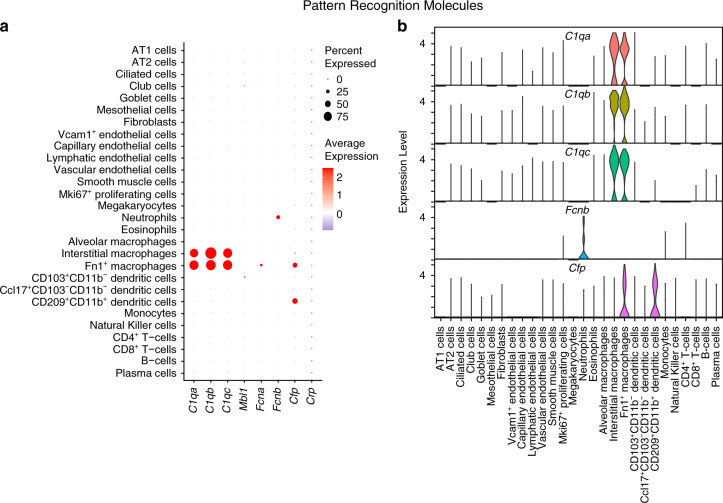
Expression profile of complement pattern recognition molecules. **a** Dot plot depicting expression of genes involved in pattern recognition in the complement system in different cell types of mouse lungs. Size of the dots represents the percentage of cells expressing the gene and color intensity represents the average expression level. **b** Violin plot of highly expressed pattern recognition genes across all cell types. Vertical lines with no violin indicate that a particular gene is expressed in only a few cells within the cell type while most cells have no expression. Sections with no violin or line indicate that the gene is not expressed in the particular cell type. Data from young C57BL/6 mice (3 months old).

Specific patterns on pathogens or damaged/infected self-cells including carbohydrate chains (non-self), pathogen associated molecular patterns (PAMPs), damage associated molecular patterns (DAMPs), clustered antibodies and short-chain pentraxins such as C-reactive protein (CRP) or serum amyloid P (SAP) can attach to the surface of suspicious cells and trigger complement activation^24^. C1q is a member of the pattern recognition molecule family and responsible for activation of CP. A single C1q protein is composed of 3 subunits (*C1qa, C1qb* and *C1qc*) which are combined as a hexamer^17^. In the lung cells of young C57BL/6 mice, all three subunits were highly expressed in >75% of Fn1^+^ macrophages and interstitial macrophages (Fig. 3a, b; Supplementary file 1). Fn1^+^ macrophages were recently described as a macrophage subset that arises through early differentiation of monocytes^25^. Ficolin-1 (*Fcna*) and Ficolin-2 (*Fcnb*), which recognize carbohydrates and activate LP, were expressed at high levels albeit in a very small percentage of Fn1^+^ macrophages and neutrophils, respectively (Fig. 3a). Properdin (*Cfp*) binds to PAMPs and DAMPs, initiates AP and enhances AP activation by stabilizing the AP C3/C5 convertases^26^. *Cfp* was highly and selectively expressed in <40% of Fn1^+^ macrophages and CD209^+^CD11b^+^ dendritic cells in the lungs of young mice (Fig. 3a, b). *Crp*, which activates the complement system via CP, showed no detectable expression in the mouse dataset.

### Proteases

**Fig. 4:**
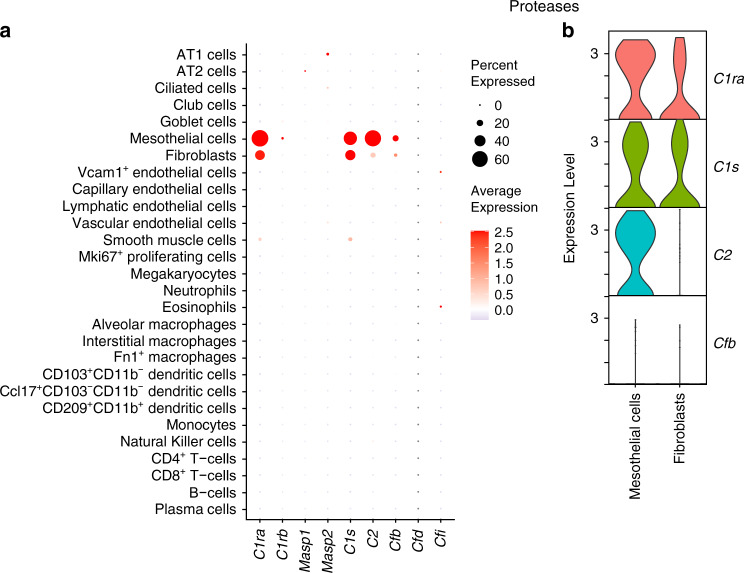
Expression profile of complement proteases. **a** Dot plot depicting expression of genes encoding proteases in the complement system. **b** Violin plot showing differential expression of the proteases in selected cell types that showed highest expression levels in **a**. Data from young C57BL/6 mice (3 months old).

Serine proteases help initiate, propagate, and regulate the complement pathway, and their activation occurs sequentially with each protease activating another. Association of pattern recognition molecules (CP, LP) or complement component C3 (AP) with zymogen proteases initiates the complement pathway^19^. Of the nine complement serine proteases examined, we found high expression of Complement component 1, r subcomponent A (*C1ra*), Complement component 1, s subcomponent (*C1s*), and Complement component 2 (*C2*) in the lung cells of young mice (Fig. 4; Supplementary file 1). Complement factor B (*Cfb*) was expressed at comparatively lower levels. These proteases function in the CP (*C1ra, C1s*), AP (*Cfb*) and CP/LP (*C2*)^19^. In addition, expression of these genes was observed only in non-immune cells, specifically fibroblasts (*C1ra, C1s*), and, interestingly, mesothelial cells (*C1ra*, *C1s*, *C2*; Fig. 4; Supplementary Figs. 4a and 5a). In fact, all three proteases showed the highest expression in mesothelial cells. Other serine proteases that function in CP (Complement component 1, r subcomponent B [*C1rb*]), LP (Mannan binding lectin serine peptidase 1 and 2 [*Masp1, Masp2*]), and AP (Complement factors d and i [*Cfd*, *Cfi*]*)* showed no detectable expression in any cell types in the mouse lung dataset.

### Complement components

In addition to *C1* and *C2*, the soluble complement components *C3*, *C4, C5, C6*, *C7*, *C8* and *C9* are critical for complement activation and terminal effector functions. C3, C4 and C5 are cleaved into two fragments (a and b). The smaller fragments (C3a and C5a) are powerful anaphylatoxins^19^. C3b can act as an opsonin and serve as the C3 convertase (AP) or C5 convertase (CP, LP and AP). C4b is part of both C3 (C4b2b) and C5 (C4b2b3b) convertases in CP and LP. In humans, mice, and rats, the *C4* gene exists in isotypic forms, chiefly *C4a* (acidic) and *C4b* (basic) with specific functions such as immunoclearance and hemolysis^27^. In the following text, *C4a* and *C4b* refers to the isotypes of the *C4* gene, rather than the cleavage product of C4 protein. C5b triggers the terminal complement pathway wherein binding of C5b to C6 initiates formation of membrane attack complex (MAC) by components C5b, C6, C7, C8 and C9, leading to lysis of the target cell. C8 encodes a trimeric complex comprising C8a, C8b and C8g subunits (as described in humans^28^).

**Fig. 5:**
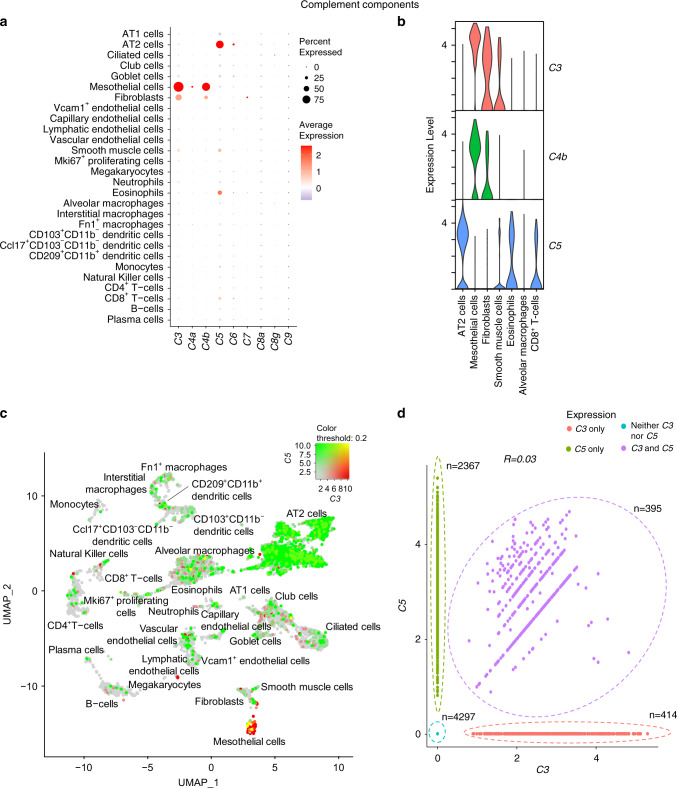
Expression profile of complement component genes. **a** Dot plot depicting expression of genes encoding complement components *C3–C9*. **b** Violin plot showing selected cell types with differential expression of complement component genes. **c** Uniform Manifold Approximation and Projection (UMAP) plot illustrating differential expression of *C3* and *C5* in lung cells. Clusters expressing *C3* are colored red, *C5* colored green, clusters expressing neither are colored grey and clusters co-expressing both are colored yellow. Intensities of the colors for each gene vary from light to dark depending on the expression level (low to high). **d** Scatter plot showing expression of C3 and C5 across the cell types. Pearson’s correlation coefficient (*R*) at the top of the plot indicates the extent of correlation between the genes. Number of cells expressing the gene(s) or not expressing either gene are indicated. Data from young C57BL/6 mice (3 months old).

Both *C3* and *C4b* were highly expressed in a large percentage of mesothelial cells (Fig. 5a, b; Supplementary file 1), with lower expression in fibroblasts (<50% of cells) of young mice. *C5*, on the other hand, was highly expressed in ~75% of AT2 cells, and at lower levels in many other cell types, predominantly eosinophils, smooth muscle cells and CD8^+^ T-cells (Fig. 5a, b). Of note, *C3* and *C5* were largely not co-expressed in the same cell types (Fig. 5c), and only detected together in *n* = 395 (~5%) cells of the entire dataset (Fig. 5d). The expression of the other complement components, namely *C4a*, *C6, C7*, *C8* (*C8a* and *C8g*) and *C9*, was low in the mouse dataset (Fig. 5a). *C8b* expression was not detectable at all in these mice (data not shown).

### Receptors

**Fig. 6:**
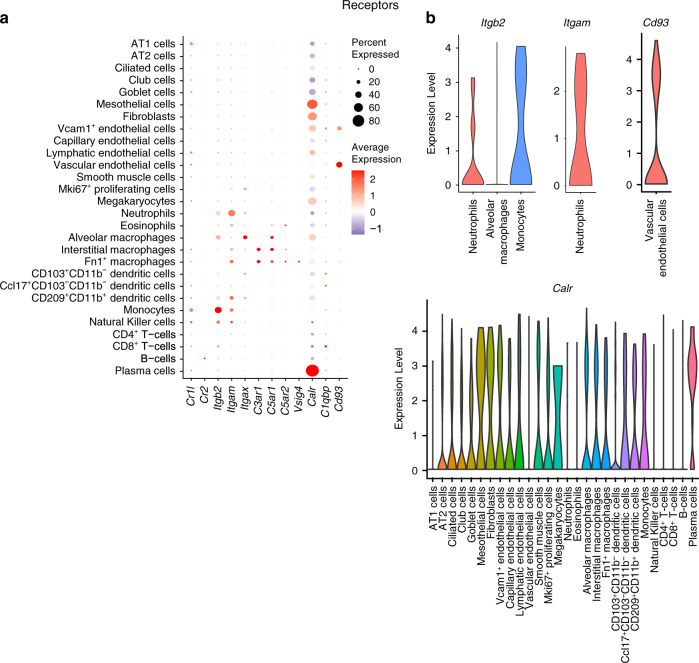
Expression profile of complement receptor genes. **a** Dot plot showing expression of receptor genes in different cell types. **b** Violin plot highlighting distribution of highly expressed receptor genes in various cell types. Data from young C57BL/6 mice (3 months old).

Complement receptors are a diverse group of proteins that play key roles in inflammatory responses through leukocyte recruitment and formation of phagosomes^19^. The complement receptors we studied included complement component (3b/4b) receptor 1-like (*Cr1l* in mice, *CR1* in humans), Complement receptor 2 (*Cr2)*, *CR3* (complex of integrins *Itgam* [CD11b] and *Itgb2* [CD18]), *CR4* (integrins *Itgax* [CD11c] and *Itgb2* [CD18]), Complement component 3a receptor 1 (*C3ar1)*, Complement component 5a receptors 1 and 2 (*C5ar1*, *C5ar2)*, V-set and immunoglobulin domain containing 4 (*Vsig4/*CRIg), Calreticulin (*Calr/*cC1qR), Complement C1q binding protein (*C1qbp/*gC1qR), and the Complement component 1q receptor 1 (*Cd93/*C1qRP)^19^. *Itgb2* was highly expressed in ~40% of the monocytes, and at lower levels in neutrophils and alveolar macrophages of young mice (Fig. 6a, b; Supplementary file 1). *Itgam* was expressed in the rare population of neutrophils at low levels and in <20% of monocytes, CD209^+^CD11b^+^ dendritic cells, interstitial macrophages and Fn1^+^ macrophages, while *Cd93* was expressed in vascular endothelial cells and at lower levels in Vcam1^+^ endothelial cells (Fig. 6a, b). *Itgax* was expressed in ~20% of alveolar macrophages (Fig. 6a). Calreticulin (*Calr*) was more ubiquitous, with high expression observed in ~60–80% of mesothelial cells, plasma cells and nearly 50% of fibroblasts (Fig. 6a, b). Comparatively fewer percentages of other cell types such as AT2, alveolar macrophages, megakaryocytes and Vcam1^+^ endothelial cells also weakly expressed *Calr* (Fig. 6). Of the anaphylatoxin receptors, *C3ar1* expression was high in <20% of interstitial and Fn1^+^ macrophages, *C5ar1* in <20% of alveolar, interstitial and Fn1^+^ macrophages while *C5ar2* was hardly detectable in any of the studied cell types. *Cr1l* was expressed in <20% of monocytes (Fig. 6a). Overall, expression of receptors trended more towards immune cells when compared with non-immune cells (Supplementary Figs. 4a and 5a), although this was only statistically significant for *Itgb2* and *Itgax* (adjusted *p* < 0.05; Supplementary file 2).

### Regulators

**Fig. 7:**
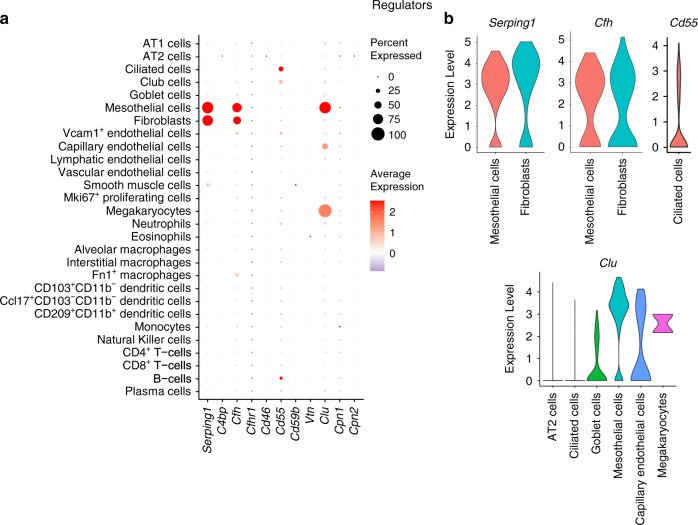
Expression profile of complement regulator genes. **a** Dot plot showing expression pattern of regulator genes. **b** Violin plot illustrating the cell-specific distribution of highly expressed regulator genes. Data from young C57BL/6 mice (3 months old).

Complement regulators modulate complement activation, persistence, and function either through inhibition of complement proteases, or competitive binding to pattern recognition molecules and complement convertases^19^. Among the different regulators of the complement system studied, C1-inhibitor (C1-INH/*Serping1*), Factor H (*Cfh*), and Clusterin (*Clu*) were highly expressed in the lungs of young mice (Fig. 7a, b; Supplementary file 1). Expression of *Serping1*, *Cfh* and *Clu* was observed in at least 70% of the mesothelial cells; *Serping1* and *Cfh* were also expressed in fibroblasts. *Clu* was also expressed in goblet cells, capillary endothelial cells, and megakaryocytes. Decay-Accelerating Factor (*Cd55*/DAF) was detected in a few ciliated cells and club cells (Fig. 7a, b). Expression of regulator genes was not detectable in most of the immune cell types annotated in young mice (Supplementary Fig. 4a). Other regulators such as Membrane Cofactor Protein (*Cd46*/MCP), Complement Component 4 Binding Protein (*C4bp*), Complement Factor H Related 1 (*Cfhr1*), Membrane Attack Complex Inhibition Factor (*Cd59b*), Vitronectin (*Vtn*), Carboxypeptidase-N (*Cpn1*, *Cpn2*) and Carboxypeptidase-B2 (*Cpb2*; data not shown) did not show any relevant mRNA expression in the lung cells identified in the murine dataset.

### Complement expression in old mice

**Fig. 8:**
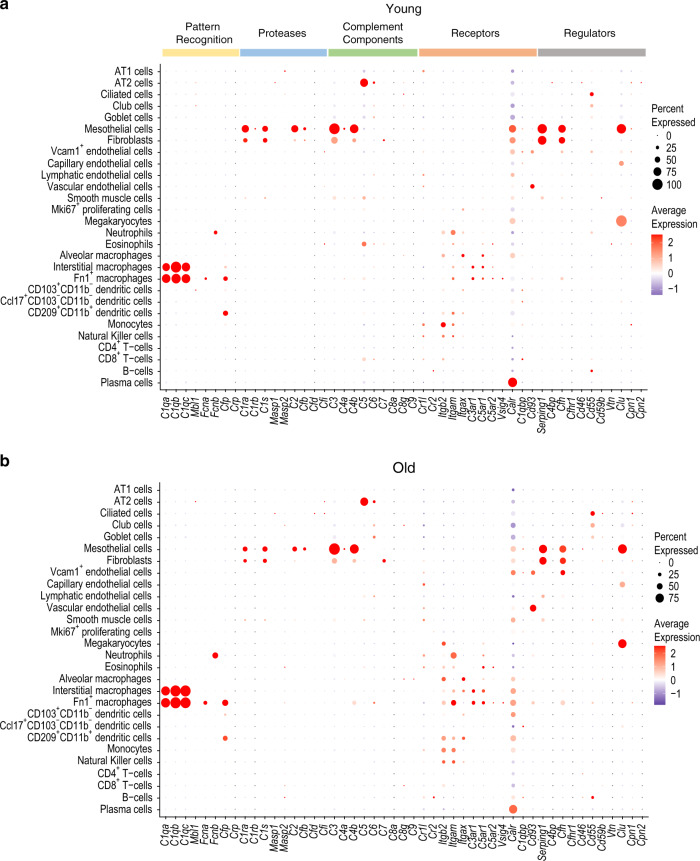
Comparison of complement genes in the lungs of young and old C57BL/6 mice. Dot plot illustrating the expression pattern of all complement genes in various cell types identified in the single cell transcriptome data for (**a**) young mice (aged 3 months, *n* = 8). **b** Old mice (aged 24 months, *n* = 7). Size of the dots represents the percentage of cells expressing a gene and color intensity represents the average expression level. Panel ‘**a**’ was prepared from the same raw data analyzed for Figs. 2–7.

A comparative analysis of expression trends of the pattern recognition molecules between old and young mice showed much more overlap than differences, with some notable exceptions (Fig. 8; Supplementary Figs. 4 and 5). Complement component *C5* appeared even more strictly confined to AT2 cells of old mice (Fig. 8; Supplementary Fig. 5). Interestingly, *C5* expression was not detected in eosinophils and CD8^+^ T-cells in old mice in contrast to young mice (adjusted *p* < 0.05; Supplementary file 3). There were also minor differences in expression profiles of complement receptors *Itgb2* and *Itgam* between old and young mice, though these differences were not statistically significant. *Itgb2* expression was comparatively higher in alveolar macrophages, Fn1^+^ macrophages, interstitial macrophages, CD209^+^CD11b^+^ dendritic cells, eosinophils, and CD4^+^ T-cells in old mice versus young mice. *Itgam* was expressed in a greater number of neutrophils, Fn1^+^ macrophages, monocytes, and Natural Killer cells from old mice versus young mice (Fig. 8; Supplementary Fig. 4). Expression of complement regulator *Cfh* was relatively higher in Vcam1^+^ endothelial cells in old mice compared with young mice (Fig. 8, adjusted *p* > 0.05; Supplementary file 3). The interpretation of age-dependent complement gene expression in lungs may need to take into consideration that differences in numbers of some cell populations existed. For example, the samples from old mice contained fewer AT2 cells and alveolar macrophages, but higher numbers of T-cells and B-cells (Supplementary Fig. 1j).

### Complement expression in human lungs

**Fig. 9:**
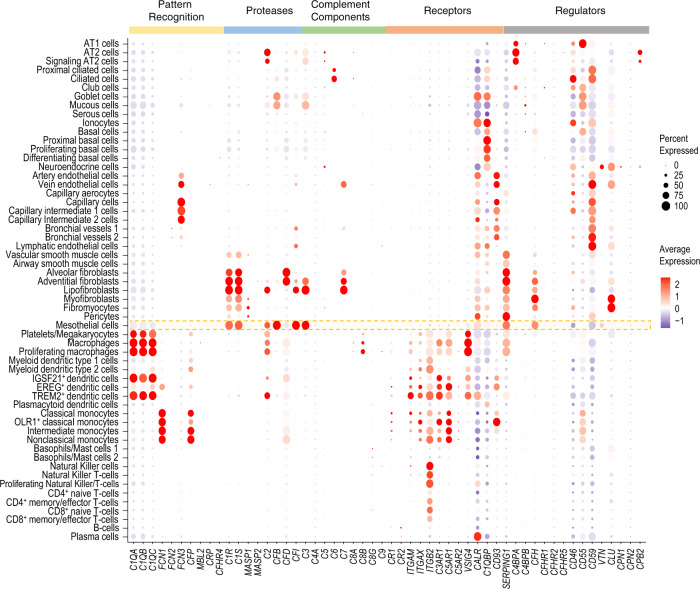
Complement gene expression in healthy human lung tissues. Dot plot illustrating the expression pattern of all complement genes in various cell types annotated in the single cell transcriptome data of healthy lung tissue of human patients (*n* = 3). Size of the dots represents the percentage of cells expressing a gene and color intensity represents the average expression level. The data is based on scRNA-sequencing from healthy, uninvolved lung regions of patients undergoing lobectomy for pulmonary tumors. Complement gene expression in mesothelial cells has been highlighted in orange.

To understand the similarities and differences in complement expression between mice and humans^29^, we also analyzed a human lung dataset generated by Travaglini et al.^3^ This dataset comprised of 57 distinct cell types as reported earlier (Supplementary Fig. 6).^3^ There were similarities and a few differences in the expression patterns of complement genes between human and mouse lungs. The most striking similarity was the high expression of several complement genes in the mesothelial cells (Fig. 9; Supplementary file 4), as observed in mouse lungs (Fig. 8). In human lungs, *C1QA–C* were expressed additionally in dendritic cells and platelets/megakaryocytes and *CFP* in monocytes. Interestingly, we observed expression of *FCN1* and *FCN3* in human monocytes and endothelial cells (including capillary intermediate cells), respectively (Fig. 9), while they were hardly detectable in the mouse lungs. High expression of *C1R*, *C1S* and *C2* was observed in human mesothelial cells and certain fibroblast sub-types (Fig. 9), quite similar to mice (Fig. 8). Moreover, human *C2* expression was also identified in macrophages, TREM2^+^ dendritic cells and platelets/megakaryocytes (Fig. 9). In contrast to mice, *CFI* was expressed highly in human mesothelial cells and lipofibroblasts, and at lower levels in a few epithelial and endothelial cell sub-types. *C3* was expressed at high levels in human mesothelial cells and lipofibroblasts, with lower expression in adventitial fibroblasts, goblet cells, mucous cells and AT2 cells. Expression patterns of complement components *C4*–*C8* were different between human and mouse lungs (Figs. 8 and 9). *C4B* was not detectable in human lungs (data not shown). *C5* was expressed in ~25% of AT2 cells, which was a lower percentage than for mouse AT2 cells. *C3* and *C5* were only detected together in *n* = 741 (~1%) cells of the entire dataset (Supplementary Fig. 7). *C6* was detectable in human ciliated cells and *C7* in human fibroblast clusters, vein endothelial cells and bronchial vessels 1. *C8B* was expressed in 25–50% of cells in the macrophage clusters (Fig. 9; Supplementary file 4). The complement receptors *ITGAM*, *ITGAX*, *ITGB2*, *C3AR1*, *C5AR1*, *VSIG4* were chiefly expressed in the myeloid cell clusters. *ITGB2* was also detectable in lymphocyte clusters, including subtypes of Natural Killer cells, CD4^+^ T-cells and CD8^+^ T-cells. *CALR* expression was ubiquitous with highest levels observed in endothelial, goblet and plasma cells, ionocytes, and some of the mesenchymal clusters. *C1QBP* was expressed mostly in goblet cells, ionocytes, basal cells, endothelial cells, lipofibroblasts, fibromyocytes, and plasmacytoid dendritic cells. *CD93* was expressed in endothelial cell clusters, similar to mouse lungs (<50% of cells; Fig. 8; Supplementary file 1), dendritic cells and monocytes; however, *CD93* was more abundant in human lungs (>50% of cells; Fig. 9; Supplementary file 4). The complement regulator *SERPING1* was expressed abundantly in mesenchymal cell clusters including mesothelial cells and fibroblasts, and at lower levels in the macrophage clusters. *C4BPA* showed high expression in ~60% of AT1, >70% of AT2 and signaling AT2 cell clusters, and ~50% of club cells (Fig. 9; Supplementary file 4). Expression of *CFH* was prominent in the human mesothelial and fibroblast cell clusters (Fig. 9), similar to mouse lungs (Fig. 8). *CD46*, *CD55* and *CD59* appeared more ubiquitously expressed in human lungs, with high levels observed in epithelial and endothelial cell clusters. Approximately 50% of human neuroendocrine cells expressed *VTN*. *CLU* was highly expressed in human myofibroblasts, fibromyocytes, neuroendocrine cells and vein endothelial cells (Fig. 9). Interestingly, *CPB2* was expressed in 30–50% of human AT2 cell clusters, whereas it was not detectable in mouse lungs (data not shown). Notably, ionocytes, which were not present in the mouse dataset, showed high expression of *CALR*, *C1QBP*, *CD46* and *CD59* (Fig. 9). Capillary aerocytes, a recently described specialized endothelial cell sub-type involved in diffusion at the gas exchange surface and leukocyte migration in the lung^6^, displayed low expression of *CD59*, *CD46*, *CALR* and *SERPING1*.

In conclusion, the dataset from three human patients recapitulated major findings in mice in terms of expression of several complement proteins in mesothelial cells and *C5* in AT2 cells, although differences for some cell types and complement genes were also noted.

## Discussion

In this study, we present a comprehensive profile of the localized expression of complement genes using bioinformatics analysis of open access mouse and human scRNA-Seq atlases. The datasets included 28 different cell types from mouse and 57 cell types from human lungs. Our results highlight expression of complement pattern recognition molecules, proteases, components, receptors, and regulators by different cell types in the lung, encompassing both immune and non-immune cells. These findings support the presence of localized, tissue-specific complement proteins in the lungs during inflammation and indicate a potential role for their non-canonical functions through molecular crosstalk with other pathways, and cell-specific homeostasis.

An interesting observation from our analysis is the expression of several proteases, complement components, receptors, and regulators at mid-to-high levels in the mesothelial cell cluster in both mice and humans. Mesothelial cells form a border surface and play an important role in both innate and adaptive immunity of the pleural compartment^9^. A study in patients with tuberculosis pleurisy reported that complement activation in the mesothelial cells might lead to increased stimulation of chemokines and monocyte chemotaxis into the pleural space contributing to disease pathophysiology^30^. Expression of mouse and human complement proteases (*C1R*, *C1S*, *C2*), components (*C3*) and regulators (*SERPING1*, *CFH*, *CLU*) in steady-state mesothelial cells supports their dual role to fuel local complement activation against invading pathogens but limit unwanted complement-mediated damage to host cells.

Another key finding from our analysis is that *C3* and *C5* are highly expressed by distinct cell types in the homeostatic state. *C3* was detected primarily in mesothelial cells and fibroblasts in murine and human lungs, as well as goblet, mucous, club and AT2 cells in humans. *C5* was mainly detected in AT2 cells. Lung fibroblasts play an important role in host immune responses through production of cytokines and lipid mediators. Intracellular expression and non-canonical cleavage of *C3* in fibroblasts might be a mechanism for homeostasis and rapid cell-specific responses to inflammation. Studies of steady-state conditions suggest that localized pools of *C3* have a role in cellular processes such as cell cycle and gene regulation^31^, and serve as reservoirs that are activated by intracellular proteases (e.g., cathepsins)^32^. C3 expression has also been observed in human alveolar macrophages, fibroblasts, B-cells, monocytes, neutrophils, epithelial, endothelial cells, and mouse AT2 cells^33,34^, highlighting similarities and differences in expression patterns compared to our analysis. Constitutive C5 expression might facilitate the immunoregulatory and repair functions of AT2 cells^35^, with levels regulated during infection and lung injury. Intracellular expression of C5 may play a role in inflammasome assembly, wherein activation of cells results in increased production of anaphylatoxin C5a^36^. This in turn stimulates C5aR1 and subsequently induces reactive oxygen species (ROS), TNFα and IL-1β production, associated with NLRP3 inflammasome assembly^32,36^. A similar role has been ascribed to C3 in NLRP3 inflammasome activation^32,36^.

A previous study reported human Properdin (*CFP*) expression in epithelial cells^16^ and alveolar macrophages^37^. However, we observed *CFP* expression only in murine Fn1^+^ macrophages, CD209^+^CD11b^+^ dendritic cells as well as human monocytes, dendritic cells, and platelets/megakaryocytes. In healthy mice, Ficolin expression was detected in neutrophils, macrophages, and lymphocytes, through western blotting^38^. Although RNA expression of *Fcna* and *Fcnb* in Fn1^+^ macrophages and neutrophils, respectively, supports the previous findings from mice, their expression was limited to a few cells in our analysis. These differences might be due to variations in transcriptional and translational rates^39^ and, possibly, sparsity in single cell data^40^. Human *FCN3* does not have an ortholog in mouse and was mainly expressed in the endothelial cell clusters. The high expression of *C1qa*, *C1qb* and *C1qc* in mouse interstitial macrophages and Fn1^+^ macrophages corroborate other murine lung studies^41^. Expression of *C1QA–C* in human lung cells has not been well characterized, though expression has been reported in macrophages and dendritic cells similar to our analysis^42^.

While high expression of the C1 inhibitor, *Serping1*, has been reported earlier in the normal mouse lung^43^, its cellular sources have not been characterized. We observed *Serping1/SERPING1* expression in mouse and human mesothelial cells and fibroblasts, where it may limit unwanted complement activation in the lung interstitium and pleural space. The highest expression of Cd55/*CD55, CD46 and CD59* was detected in airway epithelial cells and/or endothelial cells, comparable to earlier reports^16,44^. *Cfh* was mainly detected in mouse and human mesenchymal cells and in lower quantities in human epithelial cells in our analysis. *Cfh* expression has not been characterized previously in lung cell types. Carboxypeptidase *N* (*Cpn1*, *Cpn2*) and *B* (*Cpb2*) were not expressed in any of the mouse cell types in our study, while human *CPB2* was detected in AT2 cell clusters. Human *CPN* and *CPB2* have been largely characterized in the plasma and pancreas, respectively, and although mouse *Cpn1* has been detected in lungs, its cellular origin was not defined^45^.

Mouse and human *Cr1l/CR1, CR3* and *CR4* expression were largely aligned with previous reports in immune cells^46^. *C3aR1* expression has been previously reported in human and murine bronchial epithelial and smooth muscle cells^47^, *C5AR1* in human alveolar epithelial cells, endothelial cells, and smooth muscle cells^48^, and murine *C5ar1* and *C5ar2* in neutrophils, eosinophils, alveolar macrophages, CD11b^+^ dendritic cells, and monocyte-derived dendritic cells^49^. We detected low mouse *C3ar1* and *C5ar1* expression, and almost negligible *C5ar2* expression, restricted to macrophages. Human *C3AR1* and *C5AR1* showed high expression in myeloid cells in our analysis. CRIg (*Vsig4*), reported earlier in murine alveolar macrophages through western blotting^50^, showed almost no expression in any mouse cell type, whereas human *VSIG4* was strongly expressed in macrophages, dendritic cells, and platelets/megakaryocytes. While not specific to the lung, the two C1q receptors, cC1QR (*CALR*) and gC1QR (*C1QBP*) were reported in human macrophages, dendritic, B-cells, T-cells, and endothelial cells^51^. C1qRP (*CD93*) has been reported largely in human non-lung endothelial cells, monocytes, and neutrophils^52^, and in murine endothelial (non-lung) and alveolar epithelial cells^53^. The variation in the expression profiles between published reports and our analysis could be due to the differences in experimental approaches, or possibly localized profiles in the lung compared to other tissues, although further studies are needed.

Given the important role that complement genes play in modulating immune responses and homeostasis, any imbalance in the complement pathway can result in deleterious effects, as reported in various lung diseases. These include the C5a-mediated cellular damage observed in acute lung injury and the highly fatal acute respiratory distress syndrome^54,55^. In both bacterial and viral pneumonia, the pathogens escape complement-mediated eradication through either inhibition of CP or AP genes^56^. C3, C5 and their receptors have also been implicated in pathogenesis of asthma, cystic fibrosis, and idiopathic pulmonary fibrosis^16,55^. In COVID-19, excessive activation of C3 and C5a have been linked to increased viral loads, coagulation, and lung injury^20,57^.

Although our study provides a comprehensive analysis of complement expression in different cell types, there exist a few limitations. Firstly, several newly identified cell types such as ionocytes were absent in the mouse dataset, which may be due to the enzymatic isolation protocol. Nevertheless, the 28 cell types that were identified in mice represent a broad range that have versatile functions. Secondly, we may underestimate the expression of some of the complement genes due to low sensitivity (low sequencing depth of the mouse dataset), sequencing bias, and lower numbers for some cell types. This is an inherent limitation in single cell sequencing technology where many factors determine the balance between sequencing depth and the number of cells being sequenced. Thus, scRNA-Seq might be less sensitive in detecting expression when compared to technologies such as RT-qPCR. RNA-based data is also observed to have low correlation with protein abundance, with studies reporting <40% correlation, ascribed to differences in regulation of transcription and translation. Hence, the expression patterns in this analysis may not necessarily reflect the functional activity of the encoded proteins in the cell types analyzed. Another limitation is that our study is based on steady state. Complement expression patterns in pathophysiological conditions may be substantially different compared to healthy lungs. Finally, we based the cell type identities in the mouse dataset largely on the Angelidis et al. publication and extant markers^23^. Due to the evolving landscape of cell type mapping in the lungs, absence of consensus on nomenclature of certain cell subtypes and relatively low numbers of some cells in the mouse and human datasets, we suggest caution while considering findings from heterogenous populations such as fibroblasts. Moreover, the mesothelial cells are either rare or not present in typical samples of lung tissue for single cell sequencing. Despite these limitations, our findings provide a comprehensive profile of cell type specific expression of complement genes that may serve as a basis for more in-depth and targeted investigations.

Recent advances in single cell technologies, including multiomics approaches that can profile the transcriptome, proteome, and epigenome together, may help in developing a more comprehensive map in future studies. Since C3 and C5 are considered the central hubs of complement activation, and dysfunction in disease, therapeutic targeting has been mainly focused on blockade of these proteins, with several drugs such as eculizumab, ravulizumab (C5), avacopan (C5aR1) approved for use, and several more drug candidates in development. Given the complex functioning and interactivity of complement genes within multiple pathways, an inclusive design strategy driven by cell-specific cues might result in novel, targeted therapies with reduced side effects. Our findings from mouse and human lungs might serve as an initial road map that can help guide the design of such therapies in the long-term future.

## Materials and methods

### Data retrieval

Raw counts and metadata for the scRNA-Seq mouse lung atlas was obtained from the NCBI Gene Expression Omnibus database (accession number GSE124872)^23^. The dataset comprised 14813 cells: 7672 cells from 8 mice aged 3 months (‘young’) and 7141 cells from 7 mice aged 24 months (‘old’). Angelidis et al.^23^ isolated single cells from the lung tissue of male and female mice through enzymatic digestion, performed red blood cell lysis, and assessed the viability of cells by counting in a Neubauer chamber. The cells (100 cells/μL) were further processed using a Dropseq protocol and were sequenced on the Illumina HiSeq4000 platform^23^. The Dropseq computational workflow was used to demultiplex the data, align the sequences to mm10 reference genome and quantify the reads to generate the raw counts matrix comprising the number of UMIs per gene per cell^23^. This raw count matrix was the input for our analysis.

For human lung atlas, data generated by Travaglini et al. was analyzed^3^. Travaglini et al. isolated and dissociated lung tissues from uninvolved regions of three patients (aged 46 years [male], 51 years [female], 75 years [male]) undergoing lobectomy for lung tumors, together with peripheral blood^3^. The cells were sequenced using 10X 3′ v2 Chromium and Smart-seq2 protocols using the Illumina NovaSeq 6000 platform. The sequences were demultiplexed, aligned to human reference genome (GRCh38.p12) and quantified using 10X Genomics CellRanger v2. The data was filtered to only include cells with greater than 500 genes and 50,000 mapped reads. The raw counts matrix was processed using Seurat and annotated as reported by Travaglini et al. using canonical markers, excluding doublets based on expression of multiple cell type markers^3^. The processed data from lungs with cell type annotations by Travaglini et al.^3^ was retrieved from cellxgene^58^ in h5ad format for our analysis. The Smart-seq2 dataset was not used for our analysis as it did not include mesothelial cells.

### Data analysis

The mouse and human datasets were analyzed using the R framework (v 4.0.2)^59^ in RStudio Server (v 1.3.1073)^60^.

#### Mouse dataset

Based on quality control analysis, the dataset was filtered to exclude low-quality cells and only cells expressing greater than 200 genes, greater than 200 UMIs/cell, and less than 15% mitochondrial genes were used for downstream analysis. To exclude any variation in the data due to cell cycle, we normalized the data using Seurat’s (v. 4.0.1)^61^ NormalizeData function and, calculated and examined cell cycle phase scores based on expression of canonical markers. The dataset was split into young and old based on age of the mice, and the data subsets were normalized and scaled separately using NormalizeData and SCTransform functions, regressing out variation due to high mitochondrial gene expression^61^. By default, SCTransform retains the top 3000 most variable genes, which were used as anchors to integrate the two datasets (young and old) for dimensional reduction and clustering. Seurat integrates datasets based on shared cell populations, correcting for any technical differences (e.g., sequencing depth and batch effects), while preserving biological heterogeneity. Linear dimensional reduction was performed through principal component analysis and the top 42 principal components (PCs) that explained the highest variance in the dataset, were used for clustering. Nearest neighbors were identified based on the K-nearest neighbor (KNN) graph using FindNeighbors function and the cells were grouped based on the Louvain algorithm using the FindClusters function, resulting in 44 clusters. Differential expression analysis was performed using the FindAllMarkers function. Cluster annotations were based on canonical markers from literature and included subsetting and re-clustering a few groups to enhance identification of cell types. In total, the dataset was classified into 28 cell types. Clusters that showed substantial expression of multiple cell type markers in a single population were identified as doublets and excluded from further analysis. Analysis using Single Cell Toolkit (singleCellTK)^62^ package estimated a very low proportion of doublets (0.6–4%) and low ambient RNA (median contamination score: <0.1) in the processed mouse dataset. Non-linear dimensional reduction was performed using Uniform Manifold Approximation and Projection (UMAP) and was used to visualize the clusters in two-dimensional space. All dot plots, violin plots and heatmaps, and differential expression analysis were based on the RNA assay.

#### Human dataset

The processed data from cellxgene was converted to Seurat file format using SeuratDisk (v0.0.0.9019)^63^. The dataset was subset to exclude cells from the peripheral blood samples. No filtering was applied for the quality metrics for this processed dataset. The data was re-normalized using NormalizeData function in Seurat and integrated across patients using Seurat wrapper for Harmony^64^. Non-linear dimensional reduction was performed using UMAP based on the 30 principal components encoding the highest variance in the data and was used to visualize the clusters in two-dimensional space. Cell type (free) annotations by Travaglini et al.^3^ were retained and used to analyze complement gene expression. Neutrophils, reported in the original dataset, were not present in the annotated cellxgene dataset that we analyzed.

### Statistical analysis

The Wilcoxon Rank Sum Test (two-sided) through Seurat’s FindAllMarkers function was used to investigate differential gene expression between clusters/cell types, with the threshold parameters set to log1p fold-change (logFC) >0.20, min.pct of 0.1 to only test genes that are expressed in at least 10% of the cells, and multiple testing using Bonferroni correction. In addition, to test statistical significance between groups of mice (old versus young; immune versus non-immune), the two-sided Wilcoxon Rank Sum Test using Seurat’s FindMarkers function was run with the default Seurat parameters of logFC threshold of 0.25 (old versus young; immune versus non-immune cells) and min.pct of 0.1. All differential analysis were based on expression levels. The threshold for significance was set at 0.05 and adjusted *p*-values < 0.05 were considered statistically significant.

## Supplementary information

Supplementary File 1

Supplementary File 2

Supplementary File 3

Supplementary File 4

Supplementary Figure

Supplementary Table

## Data Availability

The mouse dataset for Angelidis et al. can be accessed from Gene Expression Omnibus with accession number GSE124872^23^, and the human dataset from Travaglini et al.^3^ can be accessed via cellxgene^58^ or Synapse (https://www.synapse.org/#!Synapse:syn21041850).
