# Supplementary material for: A single-cell lung atlas of complement genes identifies the mesothelium and epithelium as prominent sources of extrahepatic complement proteins
Source: Mucosal Immunol. 2022 Jun 7;15(5):927–39. doi: 10.1038/s41385-022-00534-7 (PMC9173662; doi:10.1038/s41385-022-00534-7)
Supplement: Supplementary file 5 — Supplementary Figure [file 41385_2022_534_MOESM5_ESM.pdf]

A Single-Cell Lung Atlas of Complement Genes Identifies the Mesothelium and Epithelium as Prominent Sources of Extrahepatic Complement Proteins

Neha Chaudhary, PhD[1,§], Archana Jayaraman, PhD[1,§], Christoph Reinhardt, PhD[2], Joshua D. Campbell, PhD[3], and Markus Bosmann, MD[1,2,*]

[1]Pulmonary Center, Department of Medicine, Boston University School of Medicine, Boston, 02118, MA, USA.
[2]Center for Thrombosis and Hemostasis, University Medical Center Mainz, 55131 Mainz, Germany.
[3]Division of Computational Biomedicine, Boston University School of Medicine, Boston, 02118, MA, USA.

[§]These authors contributed equally.

*Corresponding Author: Markus Bosmann M.D., Associate Professor of Medicine, Pathology and Laboratory Medicine, Pulmonary Center, Department of Medicine, Boston University School of Medicine, Boston, Massachusetts, 02118, USA, Phone: +1-617-358-1225, FAX: +1-617-638-5227. email: mbosmann@bu.edu

Supplementary Figures

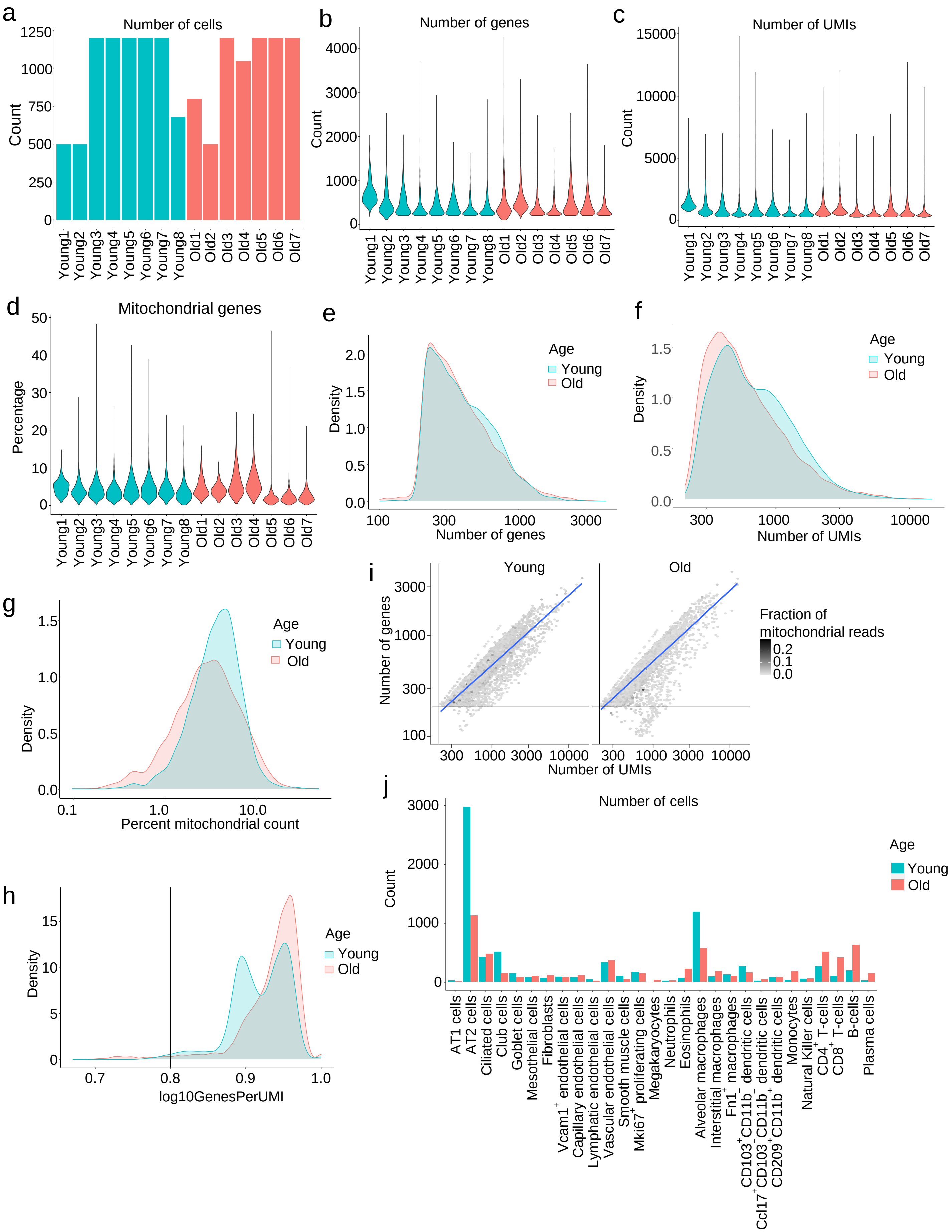

Supplementary Fig. 1: Visualization of quality control parameters and distribution of cell types. (**a**) Bar graph showing the total number of cells in each sample for young (n=8; 3 months of age) and old (n=7; 24 months of age) mice. Violin plots depicting the (**b**) number of genes, (**c**) number of Unique Molecular Identifiers (UMIs), (**d**) percentage of mitochondrial genes detected in each cell in the different samples from young and old mice. Density plots depicting the per group (young/old) distribution of (**e**) Number of genes, (**f**) number of UMIs, (**g**) percentage of mitochondrial reads per cell, (**h**) complexity as a function of number of genes per UMI. (**i**) Scatter plot of the number of genes versus the number of UMIs colored by the fraction of mitochondrial reads. (**j**) Bar graph illustrating the absolute numbers of cells in different clusters in both young and old mice. The x-axis is represented on a log10 scale for plots **e-g.**

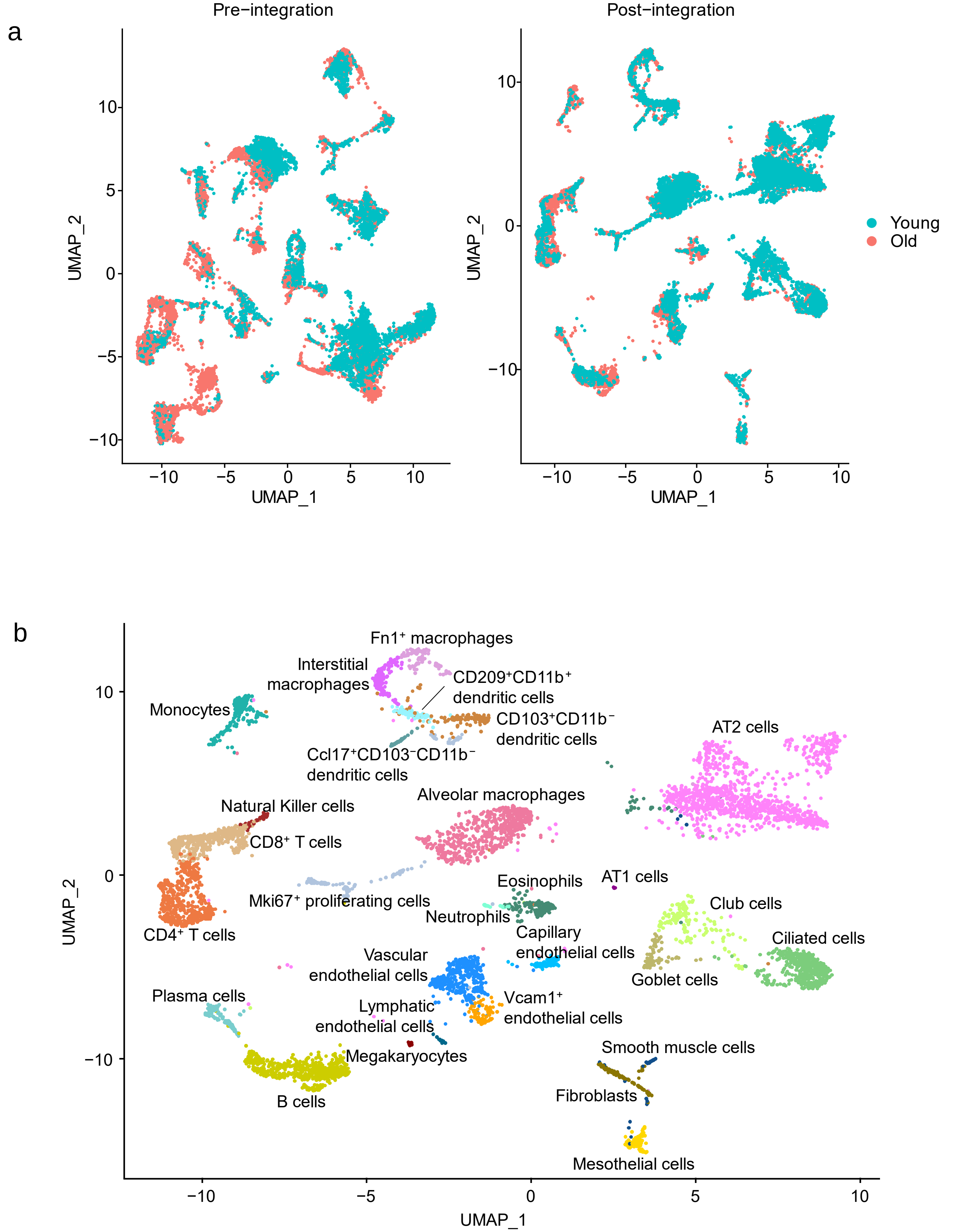

Supplementary Fig. 2: Visualization of mouse lung transcriptome. a) Uniform Manifold Approximation and Projection (UMAP) plot of young (8 mice, 3 months old) and old mice C57BL/6 mice (7 mice, 24 months old) before and after integration. Cells were clustered and annotated after integration. **b**) UMAP plot with cell type annotations of the integrated lung single cell transcriptomes from old C57BL/6 mice (n=6074 cells).

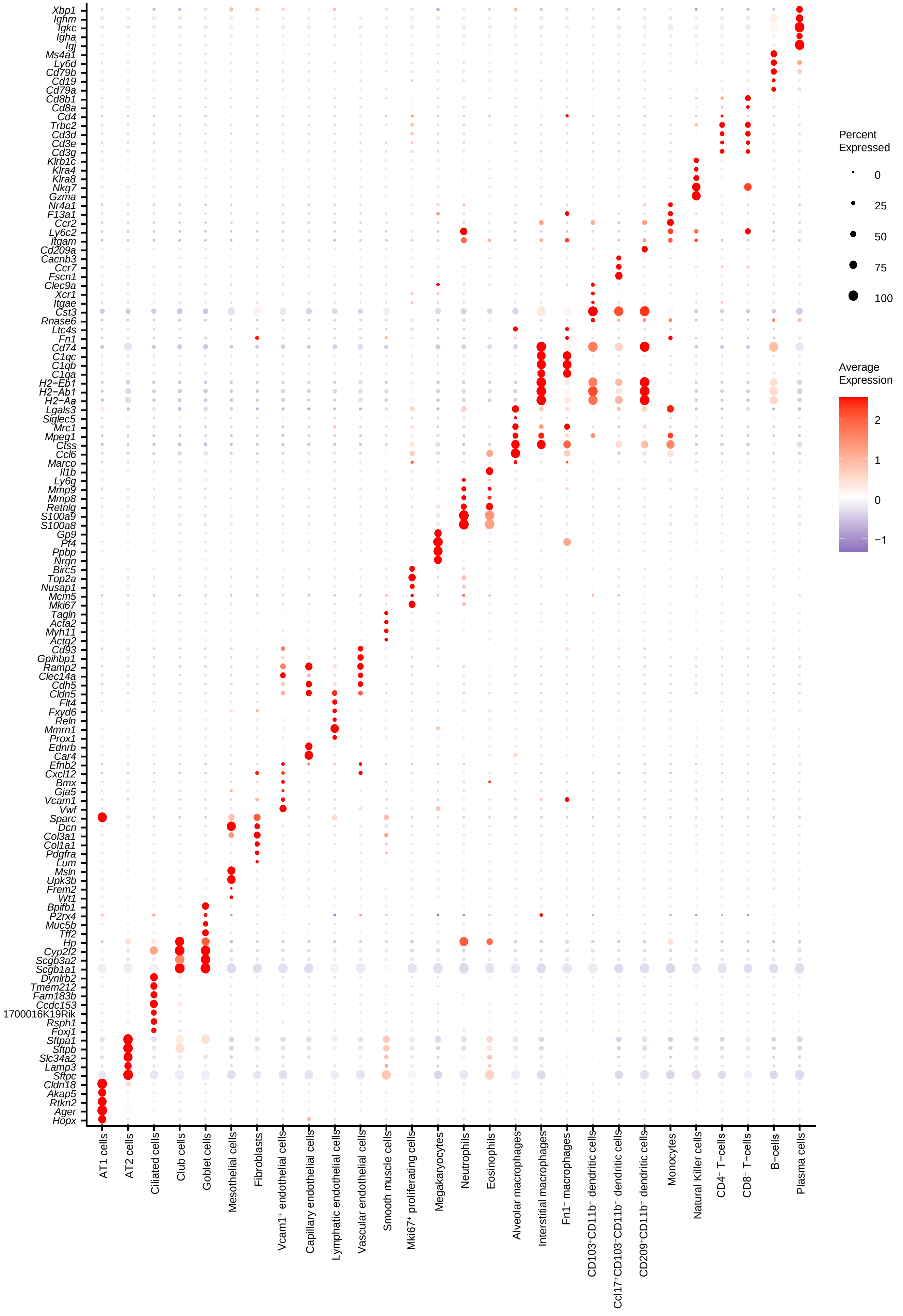

Supplementary Fig. 3: Dot plot showing the expression of selected markers used for cell type identification. Size of the dots represents the percentage of cells expressing a gene and color intensity represents the average expression level.

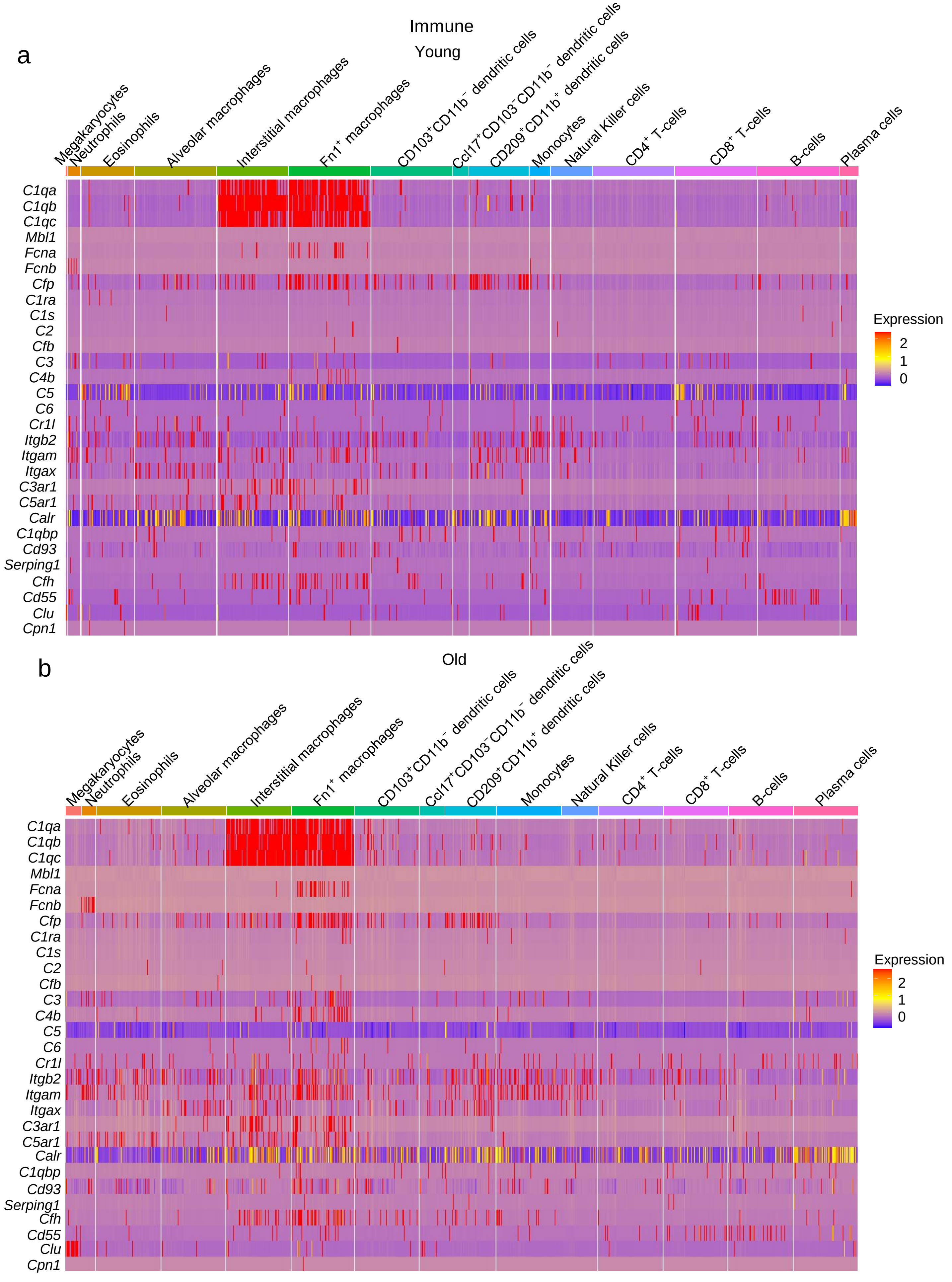

Supplementary Fig. 4: Heatmaps showing expression patterns of selected complement genes in immune cells. (**a**) young mice (n=8; 3 months of age). (**b**) old mice (n=7; 24 months of age). The data was downsampled to 100 cells (or fewer) per cell type for visualization.

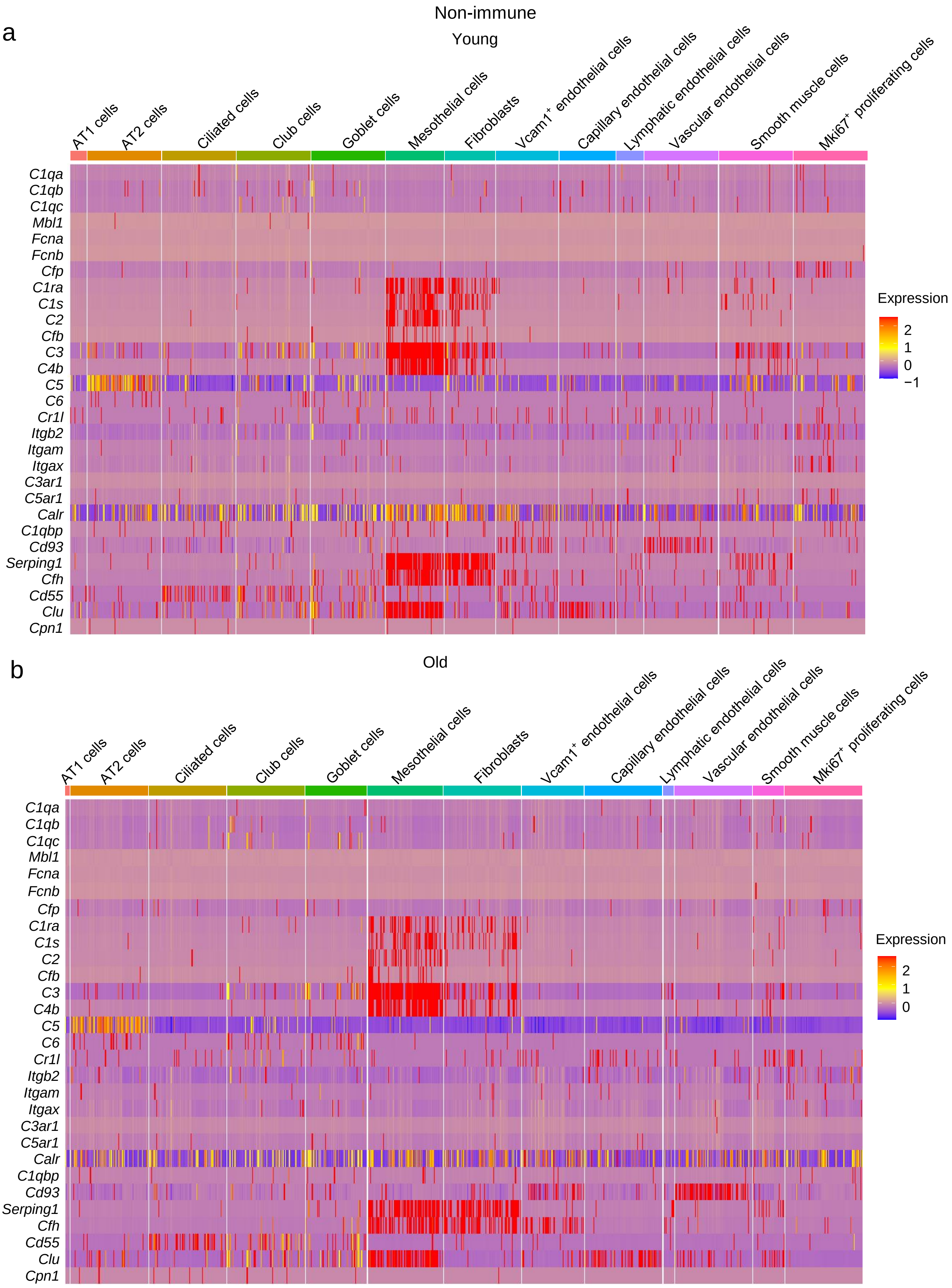

Supplementary Fig. 5: Heatmaps showing expression patterns of selected complement genes in non-immune cells. (**a**) young mice (n=8; 3 months of age). (**b**) old mice (n=7; 24 months of age). The data was downsampled to 100 cells (or fewer) per cell type for visualization.

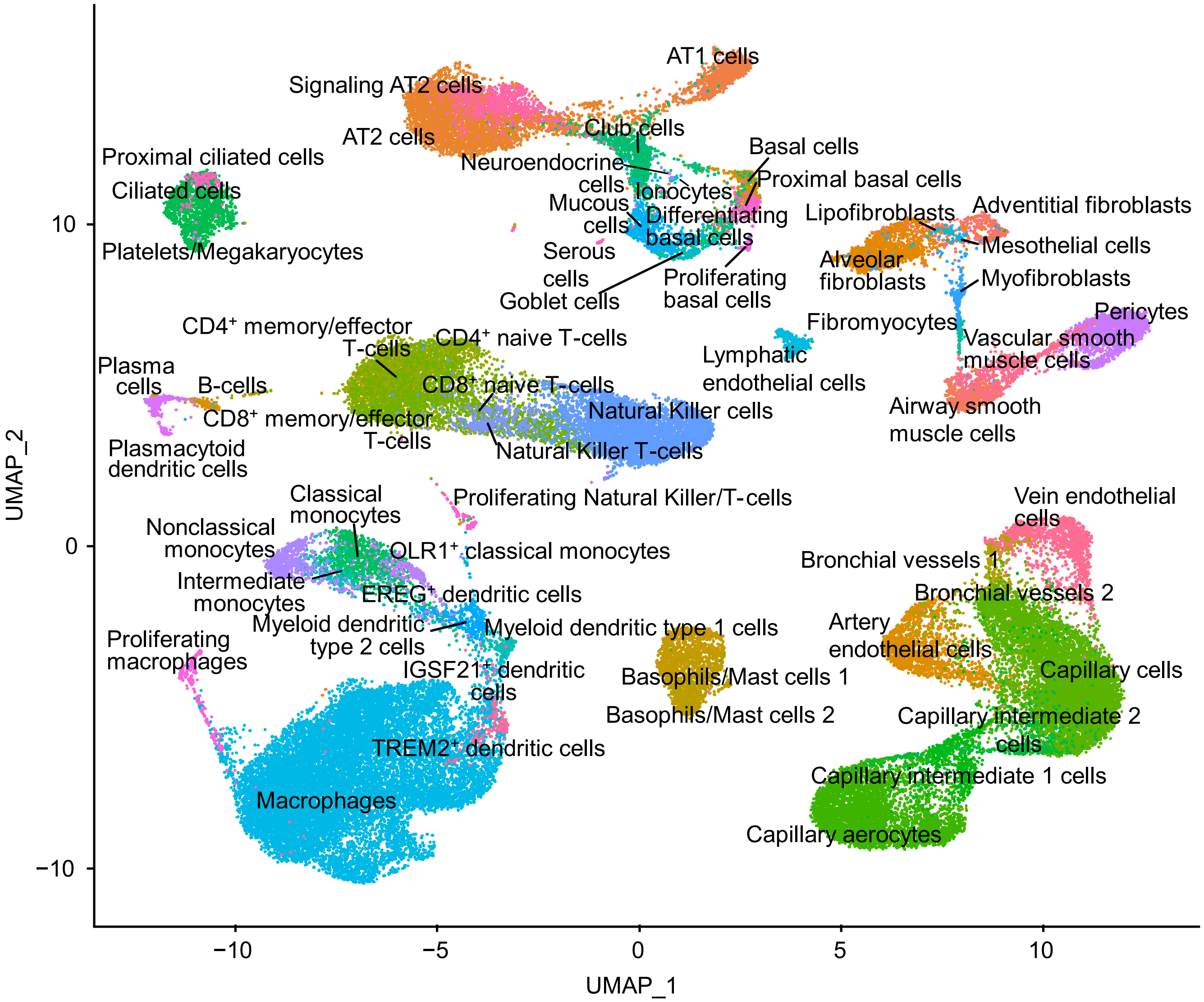

Supplementary Fig. 6: Visualization of different cell types identified in the human lung transcriptome. Uniform Manifold Approximation and Projection (UMAP) plot of lung single cell transcriptome from normal, uninvolved lobes in patients undergoing lobectomy for lung cancer (n=60993 cells from 3 individuals aged 46 [male], 51 [female] and 75 [male] years).

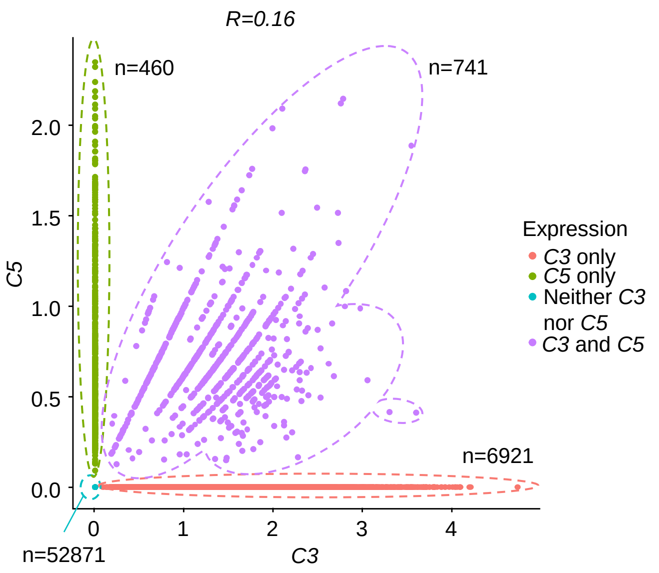

Supplementary Fig. 7: **Scatter plot showing expression of C3 and C5 across the cell types in human lung transcriptomes.** Pearson's correlation coefficient (*R*) at the top of the plot indicates the extent of correlation between the genes. Number of cells expressing the gene(s) or not expressing either gene are indicated.
