# Supplementary material for: A single-cell lung atlas of complement genes identifies the mesothelium and epithelium as prominent sources of extrahepatic complement proteins
Source: Mucosal Immunol. 2022 Jun 7;15(5):927–39. doi: 10.1038/s41385-022-00534-7 (PMC9173662; doi:10.1038/s41385-022-00534-7)
Supplement: Supplementary file 6 — Supplementary Table [file 41385_2022_534_MOESM6_ESM.pdf]

Supplementary Table 1: Nomenclature and functions of pattern recognition molecules, proteases, complement components, receptors, and regulators in *M. musculus*.[1-3]

Gene symbol	Protein	Protein name	Binding partner	Function
**Pattern recognition**				
C1qa, b, c	C1QA, C1QB, C1QC	Complement C1Q A, B, C Chains	C1R, C1S, IgG, IgM, CRP, PAMP	Initiates CP
Mbl1	MBL1	Mannose Binding Lectin 1 (Mannose-binding protein A)	MASP1, MASP2	Initiates LP
Mbl2	MBL2	Mannose Binding Lectin 2/ (Mannose-binding protein C)	MASP1, MASP2	Initiates LP
Fcna	FCNA	Ficolin A (Ficolin 1)	CRP, MASP1, MASP2	Initiates LP
Fcnb	FCNB	Ficolin B (Ficolin 2)	CRP, MASP1, MASP2	Initiates LP
Cfp	CFP	Properdin	C3bBb, C3b, C3bB	Initiates AP; stabilizes C3 and C5 convertases; senses PAMPs, DAMPs
Crp	CRP	C-Reactive Protein	C1QA, C1QB, C1QC, FCNA, FCNB, CFHR1	Senses DAMPs and PAMPs
**Proteases**				
C1ra, C1rb	C1RA, C1RB	Complement component 1, R subcomponent A, B	C1QA, C1QB, C1QC	Cleaves C1s
C1s	C1S	Complement component 1, S	C1QA, C1QB, C1QC	Cleaves C2 and C4
Masp1	MASP1	MBL Associated Serine Protease 1	MBL1, MBL2; FCNA, FCNB	Cleavage of C2, MASP2, C3
Masp2	MASP2	MBL Associated Serine Protease 2	MBL1, MBL2; FCNA, FCNB	Cleavage of C2 and C4
C2	C2a, C2b	Complement C2	After peptide cleavage of C2, fragment C2b binds to C4b	Part of C3 and C5 convertases
Cfb (B)	CFB	Complement Factor B	$C3(H_2O)$, C3	Part of the C3 and C5 convertases
Cfd (D)	CFD	Complement Factor D	CFB-C3b complex	Cleaves C3b-CFB complex to form C3 and C5 convertases
Cfi	CFI	Complement Factor I	C3b	Cleaves C3b to iC3b
**Complement components**				
C3	C3a, C3b	Complement C3	C3 cleavage peptide, C3a, binds to C3aR receptor	C3 is cleaved to form anaphylatoxin C3a and opsonin C3b; forms C3 and C5 convertases
C4 (isotypes C4a, C4b) [a]	C4a, C4b	Complement C4 (Complement C4A [Rodgers Blood Group], Complement C4B [Chido Blood Group])	After peptide cleavage of C4, fragment C4b[a] binds to C2b	Part of C3 and C5 convertases
C5 (Hc)	C5a, C5b	Complement C5	C5 cleavage peptide C5a binds to C5aR1 and C5aR2 receptors	C5 effector is cleaved to form anaphylatoxin C5a and fragment peptide C5b that initiates the membrane attack complex
C6	C6	Complement C6	C5b, C7-C9	
C7	C7	Complement C7	C5b, C6, C9, C9	
C8 (C8a, C8b and C8g trimeric complex)	C8A, C8B, C8G	Complement C8 (Complement C8 Alpha, Beta and Gamma Chains)	C5b, C6, C7, C9	Part of membrane attack complex; pore formation and lysis of pathogen cell membrane
C9	C9	Complement C9	C5b-C8	
**Receptors**				
Cr1l	CR1L (CR1)	Complement component receptor 1-like protein	C1q, iC3b, MBL	Complement regulation, clearance of opsonized molecules
Cr2	CR2	Complement receptor type 2	iC3b, C3dg, C3d	Amplifies B lymphocyte activation
Itgb2 + Itgam (Cr3)	ITGB2 + ITGAM (CR3)	Integrin Subunit Beta 2 + Integrin Subunit Alpha M complex (Complement receptor type 3)	iC3b	Induces phagocytosis
Itgb2 + Itgax (Cr4)	ITGB2 + ITGAX (CR4)	Integrin Subunit Beta 2 + Integrin Subunit Alpha X complex (Complement receptor type 4)	iC3b	Induces phagocytosis
C3ar1	C3AR1	Complement C3a Receptor 1	C3a	Immunomodulator
C5ar1	C5AR1 (CD88)	Complement C5a Receptor 1	C5a	Pro-inflammatory, immunomodulatory functions
C5ar2	C5AR2 (C5L2; GPR77)	Complement C5a Receptor 2	C5a; $C5a_{desArg}$	Pro- and anti-inflammatory functions

Supplementary Table 1: continued

Gene symbol	Protein	Protein name	Binding partner	Function
Vsig4	VSIG4 (CRIg)	V-Set and Immunoglobulin Domain Containing 4	C3b/iC3b	Facilitates phagocytosis; regulates C5 convertases
Calr	CALR (cC1qR)	Calreticulin	Collagenous stalk/tail domain of C1Q	Relays phagocytic signals
C1qbp	C1QBP (gC1qR)	Complement C1q Binding Protein	Globular head domain of C1Q	Multifunctional; phagocytic signalling
Cd93	CD93 (C1qRp)	CD93	C1Q, MBL2	Enhances phagocytosis and removal of dead cells
**Regulators**				
Serping1 (C1-INH)	SERPING1 (C1-INH)	Serpin Family G Member 1	C1RA, C1RB, C1S, MASP-1, MASP-2	inhibits CP & LP
sMAP [b]	sMAP	Small MBL-associated protein	MBL and ficolins	Inhibits LP
MAP1 [c]	MAP1	Mannose-binding lectin (MBL)/ficolin/collectin-associated protein-1	MBL, ficolins, C4b	Inhibits LP and CP
C4bp	C4BP	Complement Component 4 Binding Protein	C4bC2b → C4bp; C4b → C4c + C4d	Decay of convertases
Cfh	CFH	Complement Factor H	C3bBb → C3b + Bb; C3b → iC3b + C3f	Decay of convertases
Cfhr1	CFHR1	Complement Factor H-Related Protein 1	C5 convertase, C3b, C3d	Inactivates C5 convertase
Cd46 (MCP)	CD46	CD46 (Membrane cofactor protein)	Co-factor for Cfi (C3b → iC3b + C3f; C4b → C4c + C4d)	Regulates AP; Co-factor for Cfi
Cd55 (DAF)	CD55	CD55 (Complement Decay-Accelerating Factor)	Decay of convertases (C3bBb → C3b + Bb; C4bC2b → C4bp)	Regulates AP; Decay of convertases
Cd59b (CD59)	CD59b	CD59	C8, C9	Prevents MAC formation and apoptosis of self-cells
Vtn	VTN	Vitronectin	C5b-C9 (MAC)	Prevent cytolysis
Clu	CLU	Clusterin	C7-C9 (MAC)	Prevent cytolysis
Cpn1	CPN1	Carboxypeptidase N Subunit 1		Inactivates C5a
Cpn2	CPN2	Carboxypeptidase N Subunit 2	C5a (C5a → $C5a_{desArg}$)	Inactivates C5a
Cpb2	CPB2	Carboxypeptidase B2		Inactivates C5a

Gene and protein symbols are represented based on mouse nomenclature
[a] *C4* gene exists in two isotypic forms, *C4a* (acidic) and *C4b* (basic), while cleavage of C4 protein generates 2 peptide fragments C4a and C4b;
[b] Masp2 alternative splice product; [c] Masp1 alternative splicing product
AP, alternative pathway; CP, classical pathway; DAMP, damage-associated molecular pattern; LP, lectin pathway; MAC, membrane attack complex; PAMP, pathogen-associated molecular pattern

Supplementary References

1. Ricklin D, Hajishengallis G, Yang K, Lambris JD. Complement: a key system for immune surveillance and homeostasis. *Nat Immunol* 2010; **11**(9)**:** 785-797.

2. Kulkarni HS, Liszewski MK, Brody SL, Atkinson JP. The complement system in the airway epithelium: An overlooked host defense mechanism and therapeutic target? *J Allergy Clin Immunol* 2018; **141**(5)**:** 1582-1586.e1581.

3. Nepomuceno RR, Tenner AJ. C1qRP, the C1q receptor that enhances phagocytosis, is detected specifically in human cells of myeloid lineage, endothelial cells, and platelets. *J Immunol* 1998; **160**(4)**:** 1929-1935.
